# Cytotoxic, Antiproliferative and Pro-Apoptotic Effects of 5-Hydroxyl-6,7,3′,4′,5′-Pentamethoxyflavone Isolated from *Lantana ukambensis*

**DOI:** 10.3390/nu7125537

**Published:** 2015-12-10

**Authors:** Wamtinga Richard Sawadogo, Claudia Cerella, Ali Al-Mourabit, Céline Moriou, Marie-Hélène Teiten, Innocent Pierre Guissou, Mario Dicato, Marc Diederich

**Affiliations:** 1 Laboratoire de Biologie Moléculaire et Cellulaire du Cancer, 9 Rue Edward Steichen, L-2540 Luxembourg, Luxembourg; richard.sawadogo@lbmcc.lu (W.R.S.); claudia.cerella@lbmcc.lu (C.C.); marie_helene.teiten@lbmcc.lu (M.-H.T.); mdicato@gmail.com (M.D.); 2 Institut de Recherche en Sciences de la Santé, Ouagadougou 03 BP 7192, Burkina Faso; ip_guissou@yahoo.fr; 3 Institut de Chimie des Substances Naturelles, CNRS-ICSN UPR 2301, Université Paris-Sud, 1 avenue de la Terrasse, 91198 Gif-sur-Yvette Cedex, France; Ali.ALMOURABIT@cnrs.fr (A.A.-M.); Celine.Moriou@cnrs.fr (C.M.); 4 Laboratoire de Pharmacologie et Toxicologie de l’UFR-SDS, Université de Ouagadougou, BP 7021 Ouagadougou, Burkina Faso; 5 Research Institute of Pharmaceutical Sciences, Seoul National University, 1 Gwanak-ro, Gwanak-gu, Seoul 151-742, Korea

**Keywords:** *Lantana ukambensis*, polymethoxyflavone, cancer, anti-proliferative, cytotoxic, pro-apoptotic

## Abstract

*Lantana ukambensis* (Vatke) Verdc. is an African food and medicinal plant. Its red fruits are eaten and highly appreciated by the rural population. This plant was extensively used in African folk medicinal traditions to treat chronic wounds but also as anti-leishmanial or cytotoxic remedies, especially in Burkina Faso, Tanzania, Kenya, or Ethiopia. This study investigates the *in vitro* bioactivity of polymethoxyflavones extracted from a *L. ukambensis* as anti-proliferative and pro-apoptotic agents. We isolated two known polymethoxyflavones, 5,6,7,3′,4′,5′-hexamethoxyflavone (1) and 5-hydroxy-6,7,3′,4′,5′-pentamethoxyflavone (2) from the whole plant of *L. ukambensis*. Their chemical structures were determined by spectroscopic analysis and comparison with published data. These molecules were tested for the anti-proliferative, cytotoxic and pro-apoptotic effects on human cancer cells. Among them, 5-hydroxy-6,7,3′,4′,5′-pentamethoxyflavone (2) was selectively cytotoxic against monocytic lymphoma (U937), acute T cell leukemia (Jurkat), and chronic myelogenous leukemia (K562) cell lines, but not against peripheral blood mononuclear cells (PBMCs) from healthy donors, at all tested concentrations. Moreover, this compound exhibited significant anti-proliferative and pro-apoptotic effects against U937 acute myelogenous leukemia cells. This study highlights the anti-proliferative and pro-apoptotic effects of 5-hydroxy-6,7,3′,4′,5′-pentamethoxyflavone (2) and provides a scientific basis of traditional use of *L. ukambensis*.

## 1. Introduction

Despite considerable research efforts, cancer remains one of the most dreadful diseases in the world and the increase of its prevalence seems to be inevitable [1]. Indeed, its incidence of 12.7 million new cases in 2008 [2] is expected to rise to 21.4 million by 2030 [3]. Therefore, the search for new anticancer drugs and treatments to overcome this situation has become more and more relevant. Moreover, resistance to chemotherapy and its associated multiple side effects have forced researchers to turn increasingly towards natural products. According to Newman and Cragg, between 1981 to 2010, natural products and their derivatives were the source of 41% of new drugs and the percentage of drugs from natural products without derivatives was highly increased from 20.8% at 2009 to 50% at 2010 [4]. Each year, hundreds of various natural cytotoxic molecules were isolated or derived from medicinal plants worldwide [5,6].

**Figure 1:**
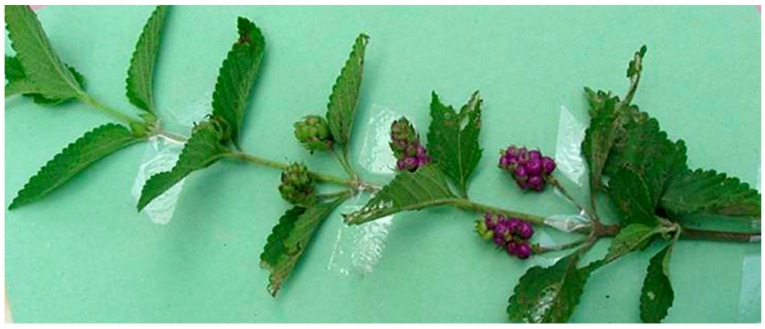
Photo of *Lantana ukambensis* (Verbenaceae) (Vatke) Verdc (Photograph by Sawadogo W.R., 2011).

In West Africa, especially in Burkina Faso, richness and diversity of the local flora is considered an inexhaustible source of new molecules [6]. Here we investigated isolated compounds from *Lantana ukambensis* (Vatke) Verdc. (Verbenaceae), a food and medicinal plant from Burkina Faso used in folk medicine to treat chronic wounds and skin diseases. In many African countries, *L. ukambensis* (Figure 1) is harvested from the wild as a local source of food, medicine, insect repellent, and for fuel [7]. Previously, we reported the significant anti-proliferative effect of that plant extracts against KB (KERATIN-forming tumor) cell lines and its anti-leishmanial property [8,9]. Moreover, preliminary phytochemical screening showed the presence of saponins, flavonoids, tannins, triterpenoids, and steroids [9]. All of these compounds are well known for their anticancer properties [10,11,12,13,14] and could be responsible for the beneficial effect of that plant. The objective of the present study was to isolate cytotoxic molecules from *Lantana ukambensis* using bio-guided fractionation and isolation methods and to elucidate their chemical structure. In addition, the cytotoxicity, anti-proliferative and apoptotic effects of the isolated molecules were carried out on cancer and healthy cells.

## 2. Materials and Methods

### 2.1. Plant Material

Sawadogo WR collected the whole plant of *L. ukambensis* (Vatke) Verdc. (Verbenaceae) in August, 2010 by Bobo Dioulasso in the west part of Burkina Faso with the following GPS position: Latitude (dd) of 11.19517 and Longitude (dd) of −4.36616. In this country, researchers have the right to collect samples of medicinal plants for the purposes of their research. The plant was identified by Professor Millogo Jeanne at the Laboratory of Ecology, University of Ouagadougou, Burkina Faso. The plant name was validated with the plant list database: http://www.theplantlist.org/tpl1.1/record/kew-108330. A voucher specimen has been deposited at the herbarium of the Centre National de la Recherche Scientifique et Technologique (CNRST) under the specimen number LU-003. The whole plant was air dried for two weeks in laboratory conditions and pulverized.

### 2.2. Extraction

50 g of *L. ukambensis* powder were macerated with stirring for 24 h in methylene chloride (250 mL). After filtration, the extract was evaporated to obtain dried extract (4.5 g) that was used for the bio-guided isolation of cytotoxic molecules.

### 2.3. Bio-Guided Isolation and Structure Elucidation

Firstly, the methylene chloride extract of *L. ukambensis* was fractionated by the Thin Layer Chromatography method using preparative TLC plates (silica gel, 500 µm, 20 × 0 cm, Sigma-Aldrich, Saint-Quentin Fallavier, France) and the following mobile phase cyclohexane/ethyl acetate/ethanol/acetic acid (6/2/2/0.1). Each TLC fraction was tested for cytotoxicity on K562 cells using trypan blue exclusion assay to highlight cytotoxic fractions. Secondly, the cytotoxic fractions were analyzed and fractionated again by High Performance Liquid Chromatography (HPLC) in order to isolate cytotoxic compounds. HPLC conditions were: Waters Sunfire C-18 semi-prep (Φ 10 mm × 150 mm, 5 µ) for the column; H2O + 0.1% formic acid/acetonitrile + 0.1% formic acid for the solvent; 70/30 to 10/90 in 20 min as gradient; 4.5 mL/min as flow rate; and PDA for the detection. Finally, the isolated compounds were tested for the cytotoxic effect against K562, Jurkat, Raji, U937, and PBMCs cells. For chemical structure elucidation, the isolated compounds were analyzed by Mass Spectroscopy (MS), Nuclear Magnetic Resonance (NMR), and comparison with literature spectroscopic data.

Compound (1) was isolated as a white amorphous solid and its structure was identified by comparison with published spectroscopic data, including ESI-mass spectrum, that showed a [M + H]^+^ ion peak at *m/z* 403 and 2D NMR spectra performed in CDCl3. All these data were in full agreement with the described compound as 5,6,7,3′,4′,5′-hexamethoxyflavone (1) [15].

Compound (2) was isolated as a white amorphous solid and its structure was identified by comparison with published spectroscopic data, including ESI-mass spectrum that showed a [M + H]^+^ ion peak at *m/z* 389 and 1H, 13C NMR and 2D NMR spectra performed in CDCl3. All these data were identical with those described for Umuhengerin (2) [16]. Purity of (1) and (2) is more than or equal to 95% as determined by HPLC and NMR.

### 2.4. Cell Culture

Human K562, Jurkat, Raji, and U937 cells were cultured in RPMI 1640 medium (Lonza, Verviers, Belgium) supplemented with 10% fetal bovine serum (Lonza), penicillin (100 units/mL), and streptomycin (100 μg/mL) at 37 °C with 5% CO_2_ and harvested every three days for maintenance. For each treatment, cells were seeded at a concentration of 300,000 cells/mL and treated after 24 h, in condition of exponential growth. Treatments were applied to exponentially-growing cells. Healthy blood samples were kindly donated as buffy coats by Red Cross (Luxembourg, Luxembourg). PBMCs were extracted as previously described [17].

### 2.5. Viability Assay

The viability assay was assessed by trypan blue exclusion test allowing determining the number of viable cells present in a cell suspension. Its principle is based on the fact that living cells possess intact cell membranes that exclude trypan blue dye while dead cells do not. Cells were seeded in 24-well plates at 200,000 cells/mL. Different concentrations of crude extract, fractions, isolated compound, or UNBS1450 (positive control) were mixed in each well, while the negative control was mixed with the same volume of DMSO. In all experiments of this study, the concentration of DMSO did not exceeded 1% to avoid significant toxicity of that solvent on tested cells. The hemi-synthetic cardiac glycoside UNBS1450 [18,19,20,21] was a kind gift from Unibioscreen (Brussels, Belgium) and its purity exceeded 98% according to the HPLC analysis. At each time course (24 h, 48 h, and 72 h), 20 μL of the cell suspension was mixed with 20 μL of trypan blue. 20 μL of this mixture was applied to a haemocytometer and observed by a binocular microscope. The viable cells were counted in negative control and treated samples and the percentage of viability was calculated from three independent tests.

### 2.6. Proliferation Assay

The evaluation of the anti-proliferative effect of (2) on U937 cells was carried out using IncuCyte TM Live-Cell Imaging System (Essen BioScience, United Kingdom). Briefly, cells were seeded in 24-well plates pre-coated with Poly-lysine D and incubated for 3 h before treatment to allow cell fixation. Cells were treated by (2) or DMSO (negative control) and Celecoxib (Sigma-Aldrich, (Bornem, Belgium) purity exceeded 98% by HPLC, Saint-Louis, MO, USA) at 7.5 µg/mL (positive control) [22,23]. Results are expressed as the percentage of confluence (viable cells) *versus* time after three independent tests.

### 2.7. Analysis of Apoptosis

The apoptotic effect of (2) was assessed using two methods: Analysis of nuclear fragmentation (Hoechst staining method) and flow cytometry analysis (Annexin V/ Propidium Iodide staining (PI) assays) as previously described [12,24]. U937 cells were cultivated in medium containing the indicated concentrations of (2) or UNBS1450 (positive control) [12,18,19,20] for 24 and 48 h. The level of apoptosis was measured by using two different approaches. Nuclear fragmentation attesting apoptosis was highlighted by DNA-specific dye Hoechst 33342 (Sigma Aldrich) and observed by fluorescence microscopy (Leica –DM IRB microscope, Lecuit, Luxembourg) and the percentage of apoptotic cells was calculated from the number of cells with nuclear apoptotic morphology in at least 300 cells in at least three independent fields [12,24]. The images were analyzed using the Image J software vIJ 1.48v (http://rsb.info.nih.gov/ij/docs/index.html). Alternatively, U937 cells were assayed for phosphatidylserine exposure at the indicated times and doses of treatment. The Annexin V-FITC apoptosis detection kit I (Becton Dickinson Biosciences, Erembodegem, Belgium) was used according to the manufacturer’s instructions. Stained samples were analyzed by FACS (FACSCalibur, Becton Dickinson, San Jose, CA, USA). Data were recorded using the BD CellQuest™ Pro software (version 4.0.2) (http://www.bdbiosciences.com/features/products) for further analysis.

### 2.8. Statistical Analysis

Results from at least three independent experiments were statistically analyzed through Student’s t-test using GraphPad Prism version 6.00 for Windows, GraphPad Software, La Jolla, CA, USA, www.graphpad.com. *p*-values below 0.05 (*) or 0.01 (**) were considered as significant.

## 3. Results

### 3.1. Bioguided Isolation of Cytotoxic Molecules

**Figure 2:**
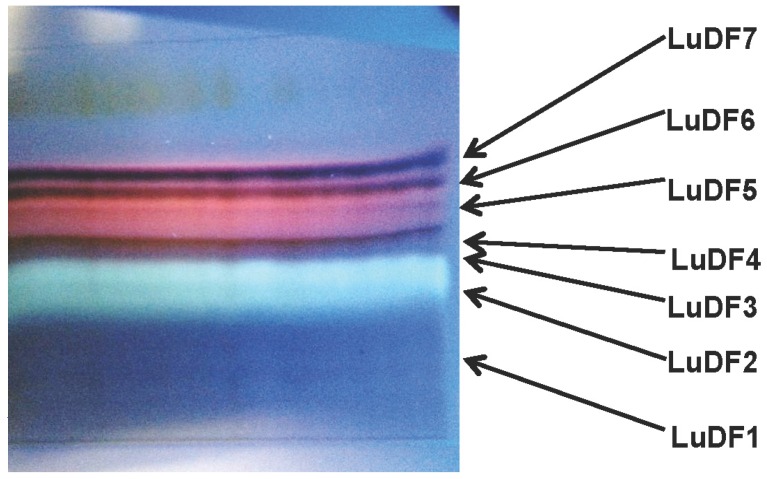
Thin layer chromatography (TLC) fractionation of a methylene chloride extract of *L. ukambensis*. Mobile phase: Cyclohexane/Ethyl acetate/Ethanol/Acetic acid (6/2/2/0.1). Plate was revealed under UV365 nm. LuDF: *Lantana ukambensis* Dichloromethane Fraction.

**Figure 3:**
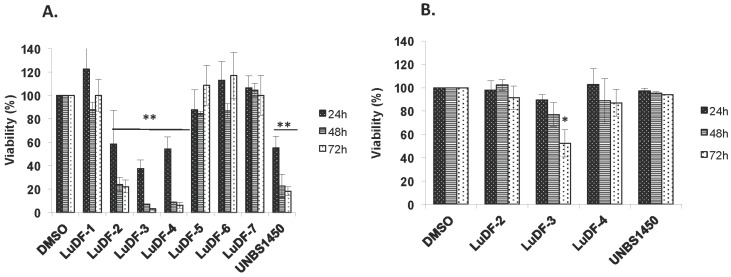
Viability assays by trypan blue exclusion test on K562 cells treated or not with TLC fractions (10 µg/mL) of *L. ukambensis* extract for 24, 48, and 72 h (**A**); cytotoxic fractions were tested on PBMCs (peripheral blood mononuclear cells) under the same conditions (**B**). UNBS1450 was used as positive control at 0.01 µg/mL. Results are the mean ± SD from three independent experiments. LuDF: *Lantana ukambensis* Dichloromethane Fraction; (DMSO): dimethyl sulfoxide.

**Figure 4:**
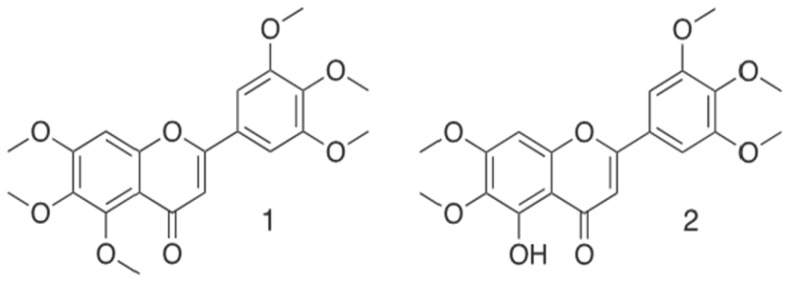
Structural formulas of isolated compounds 5,6,7,3′,4′,5′-hexamethoxy-flavone (1) and 5-hydroxy-6,7,3′,4′,5′-pentamethoxyflavone (2) from *Lantana ukambensis* (Verbenaceae).

Methylene chloride maceration of 50 g of *L. ukambensis* powder gave 4.5 g of crude extract (yield of 9%). Chromatographic fractionation on silica gel of 500 mg of the crude extract gave seven fractions; namely LuDF1 (*Lantana ukambensis* Dichloromethane Fraction 1), LuDF2, LuDF3, LuDF4, LuDF5, LuDF6, and LuDF7, with the masses of 55, 45, 48, 51, 36, 46, and 58 mg, respectively (Figure 2). Three of the seven fractions (2–4) were found to be significantly cytotoxic to K562 cells at 10 µg/mL with less toxicity against PBMCs (Figure 3A,B). The known compound, 5,6,7,3′,4′,5′-hexamethoxyflavone (1) was isolated from fraction 2 and its analogue, 5-hydroxy-6,7,3′,4′,5′-pentamethoxyflavone (2), from fraction 4 (Figure 4).

### 3.2. Cytotoxic, Anti-proliferative, and Apoptotic Effects of Isolated Molecules

**Figure 5:**
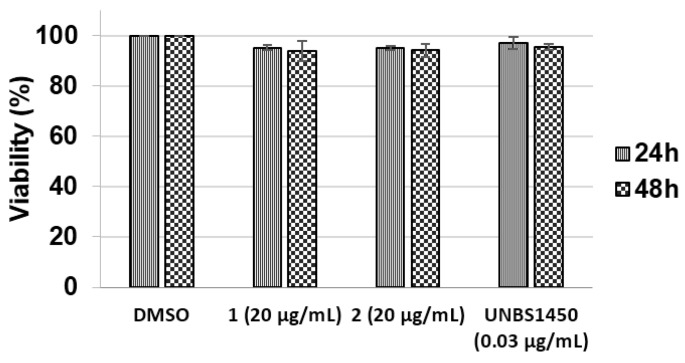
Cytotoxic effect of (1) and (2) at 20 µg/mL and UNBS1450 at 0.03 µg/mL on PBMCs (peripheral blood mononuclear cells) for 24 and 48 h. Viability was evaluated by trypan blue exclusion assay. Results are the mean ± SD (Standard Deviation) of three independent experiments.

**Figure 6:**
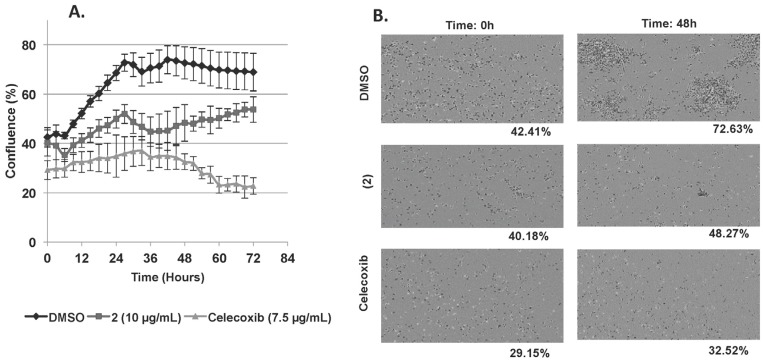
(**A**,**B**) Anti-proliferative effect of (2) at 10 µg/mL on U937 cells was evaluated by Incucyte TM Live-Cell Imaging System and is presented here as U937 cells growth curve plotting confluence *vs.* time at six hour intervals (**A**); DMSO and Celecoxib (7.5 µg/mL) were used respectively as negative and positive controls. Data are the mean ± SD (Standard Deviation) of three independent experiments with three samples per experiment. IncuCyte TM images of U937 confluence after 48 h of treatment with (2) *versus* DMSO and Celecoxib (**B**).

The two isolated analogues were tested against different cancer cell lines, including K562, Jurkat, Raji, and U937 *versus* PBMCs from healthy donors. Only (2) was found to be cytotoxic against U937, Jurkat, and K562 cells after 72 h of exposure with IC_50_ (concentration causing 50% inhibition) values of 6.1 ± 0.7, 9.6 ± 1.1 and 11.9 ± 0.4 μg/mL respectively (Table 1). Raji cells appeared resistant to this molecule (IC50 ≥ 20 µg/mL). In addition, (1) and (2) appeared non-toxic towards PBMCs at 20 µg/mL (Figure 5) whilst LuDF3 exhibited significant cytotoxicity on these healthy cells at 10 µg/mL (Figure 3B). The anti-proliferative effect of (2) was evaluated by an Incucyte Live-Cell Imaging System (Essen BioScience, St Albans, United Kingdom) on U937 cells and the results are presented as a growth curve plotting confluence *vs.* time with six-hour intervals (Figure 6A,B) (video is available as Supplementary Material S1). Compound (2) exhibited significant anti-proliferative effects against U937 cells (8.1% of proliferation after 48 h of exposure, Figure 6B) compared to dimethyl sulfoxide (DMSO) (30.2% of proliferation after 48 h of exposure). Nevertheless, the anti-proliferative effect of (2) is lower compared to Celecoxib used as a positive control (3.4% of proliferation after 48 h of exposure). Celecoxib was chosen as a control anti-proliferative compound based on our previous publications [22,23,25,26].

**Table 1:** IC_50_ values of (2) and UNBS1450 (positive control) on U937 cells at 24, 48, and 72 h.

IC_50_ Values (µg/mL)		U937	Jurkat	K562	Raji
Compound (2)	24 h	9.3 ± 1.2	23.7 ± 9.5	NA	NA
	48 h	7.7 ± 1.7	12.2 ± 5.7	20.5 ± 2.3	NA
	72 h	6.1 ± 07	9.6 ± 1.1	11.9 ± 0.4	NA
UNBS1450	24 h	0.010 ± 0.001	0.014 ± 0.001	0.033 ± 0.006	0.025 ± 0.007
	48 h	0.007 ± 0.001	0.008 ± 0.001	0.013 ± 0.005	0.014 ± 0.004
	72 h	0.004 ± 0.001	0.006 ± 0.001	0.015 ± 0.001	0.008 ± 0.001

NA: No activity.

**Figure 7:**
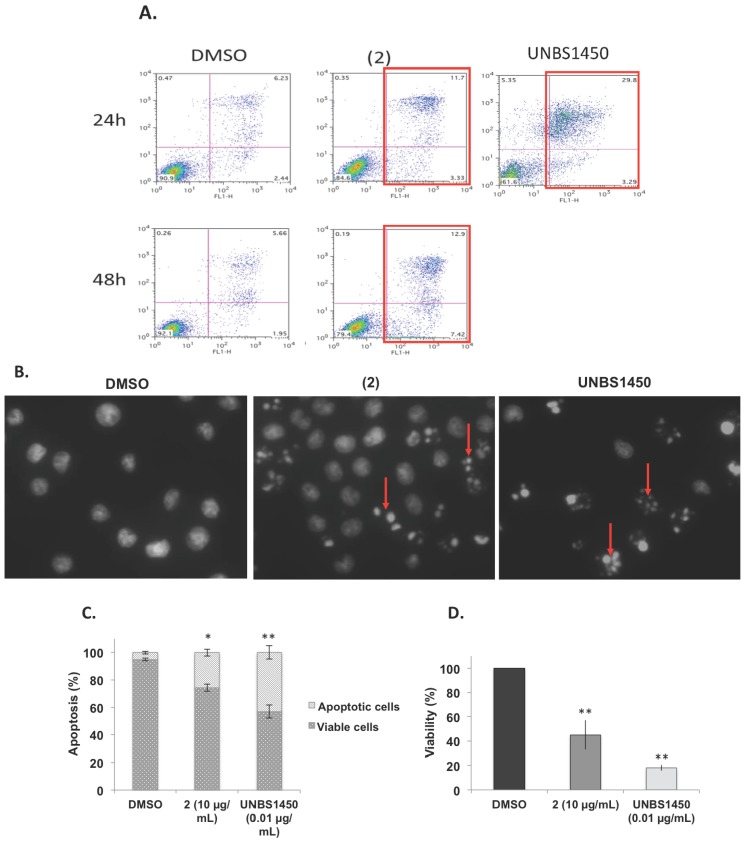
(**A**–**D**): Apoptotic effect of compound 2 at 10 µg/mL and UNBS1450 at 0.01 µg/mL, 24 h on U937 cells. U937 cells treated with (2) at 10 µg/mL, dimethyl sulfoxide (DMSO) (negative control) or UNBS1450 at 0.01 µg/mL (positive control) were subjected to an Annexin V/PI staining assay. Results were confirmed by three independent analyses, the red squares indicate the cytotoxic effect (**A**); Hoechst staining of U937 cells treated with DMSO or (2) at 10 µg/mL compared to UNBS1450. Nuclear fragmentation (indicated by red arrows) is observed in cells treated with (2) or UNBS1450 (**B**); apoptotic effect of (2) at 10 µg/mL or UNBS1450 (positive control) was evaluated by Hoechst staining assay (**C**), compared to its cytotoxicity evaluated by trypan blue exclusion test (**D**). Data are the mean ± SD (Standard Deviation) of three independent experiments.

Apoptotic effect of compound (2) was evaluated by Annexin V/PI staining and Hoechst staining assays compared to DMSO (solvent control) and UNBS1450 (control cytotoxic compound) at 24 h on U937 cells. Results were confirmed by three independent analyses; the red squares indicate the cytotoxic effect (Figure 7A). The percentage of dead cells from Annexin V/PI staining are 8.7% for DMSO, 15.0% for Compound 2 and 33.1% for UNBS1450 after 24 h of exposure. Nuclear fragmentation testifying the apoptotic effect (indicated by red arrows, Figure 7B) is observed in cells treated with (2) or UNBS1450. Nevertheless, the apoptotic effect of (2) evaluated by Hoechst staining assay (Figure 7C) is less than its cytotoxicity evaluated by the trypan blue exclusion test (Figure 7D).

## 4. Discussion

Two polymethoxyflavones were isolated from *L. ukambensis* and identified in this study. The first one, 5,6,7,3′,4′,5′-hexamethoxyflavone (1) was previously isolated in citrus reticulate by Itoh and co-workers and its inhibitory effect on histamine release in RBL-2H3 cells (rat basophilic leukemia) was reported [27]. The second compound, 5-hydroxy-6,7,3′,4′,5′-pentamethoxyflavone (2) was previously isolated from *Lantana trifolia* by Rwangabo [16] and from *Murraya paniculata* by Kinoshita and Firman [15] and exhibited anti-microbial and anti-fungal activities against various pathogens, including *Staphylococcus aureus*, *Salmonella typhimurium*, *Candida tropicalis*, *Aspergillus niger*, *Aspergillus fumigatus*, *Trichopyton mentagrophytes*, and *Microsporum canis* [16]. Nevertheless, according to our knowledge, these two compounds have never been studied for cytotoxic activity; hence the interest of our study. Only (2) was found to be cytotoxic against U937, Jurkat, and K562 cells. According to the chemical structure comparison of (1) and (2) (Figure 3), we suspect that the cytotoxic effect of (2) is due to the hydroxyl and ketone moieties in carbons 5 and 6, respectively. These moieties are well known for their antioxidant, anti-proliferative and cytotoxic properties [28,29]. In addition, substitution of the hydroxyl group in C5 by a methoxy moiety abrogates the cytotoxic effect [28,30]. Interestingly, (2) is in accordance with the Lipinski “rule of 5” for planned *in vivo* assays. According to Lipinski *et al.*, four parameters are globally associated with solubility and permeability including the number of H-bond donors and H-bond acceptors, molecular weight and Log P, leading to the “rule of 5”. That “rule of 5” states that poor absorption or permeability can be observed in compounds that contains more than five H-bond donors and 10 H-bond acceptors, with molecular weight over than 500 and Log P greater than five. Log P allows understanding the hydrophilicity or hydrophobicity (lipophilicity) of each molecule. Indeed, if the LogP is positive and very high, the compound is lipophilic, and conversely, if the Log P is negative this means that the molecule is hydrophilic. Mostly, when two parameters are out of this range, the concerned compound is more likely to lose its pharmacological properties when tested *in vivo* [31,32]. In perspective, we will investigate the structure-activity relationship (SAR) through structural modification of (2) in order to find the most active compound with less toxicity against healthy cells.

Compound (2) demonstrated for the first time its anti-proliferative and pro-apoptotic effects against U937 cells by a Live-Cell Imaging System (IncuCyte TM) and through Hoechst and Annexin V/Propidium Iodide staining (PI) assays. The nuclear fragmentation observed in Hoechst and Annexin V/Propidium Iodide staining (PI) assays are similar to those obtained with UNBS1450 [12,18,19,22,23,24]. In addition, we observed that the percentage of cell death by apoptosis (Figure 7C) was less than the impact on cell viability assessed with trypan blue exclusion assay (Figure 7D). This discrepancy may be most likely due to the fact that cells are rapidly damaged and their nuclei disintegrated, therefore they can no longer be observed by Hoechst staining. Accordingly, apoptotic cells appeared to rapidly lose plasma membrane integrity, therefore switching to a status of secondary necrosis (as highlighted by the accumulation of double-positive Annexin V/PI cells) (Figure 7A, upper-right quadrants). In perspective, we will evaluate, by Western blot, the expression of the apoptosis-related proteins including caspases, MCL-1, and XIAP in order to elucidate the pro-apoptotic mechanism of (2) in U937 cells.

## 5. Conclusions

Our results showed that (2) has a good profile for further studies because of its selective toxicity against cancer cells and its accordance with Lipinski’s “rule of 5”. According to the literature, this is the first time that (2) is isolated from *Lantana ukambensis* and that its cytotoxic activity is highlighted. Our results obtained through this study give a first scientific insight into the potential mode of action of a compound from traditionally-used *Lantana ukambensis* and provides a basis for further investigation of the cytotoxic potential of this food and medicinal plant.

## Supplementary Materials

Video S1: Anti-proliferative effect of (2) at 10 µg/mL on U937 cells was evaluated by Incucyte TM Live-Cell Imaging System (Incucyte software version 2011A Rev2) and is presented here as U937 cells growth video during 72 h. Celecoxib (Video S2) and DMSO (Video S3) were used as positive and negative controls, respectively. Supplementary materials can be accessed at: http://www.mdpi.com/2072-6643/7/12/5537/s1
